# Intraoperative Methadone for Postoperative Pain Control in Living Donor Hepatectomy

**DOI:** 10.1155/joot/8206764

**Published:** 2026-09-09

**Authors:** Adam Seibert, Devika Puri, Zachary Whong, Beomchang Kim, Rasha Alsaadawi, Le Kang, Christin Kim, Seung Duk Lee, Vinay Kumaran, Smita Pancholia, Sergio Navarrete

**Affiliations:** ^1^ Department of Anesthesiology, Virginia Commonwealth University, Richmond, Virginia, USA, vcu.edu; ^2^ School of Medicine, Virginia Commonwealth University, Richmond, Virginia, USA, vcu.edu; ^3^ Department of Biostatistics, School of Public Health, Virginia Commonwealth University, Richmond, Virginia, USA, vcu.edu; ^4^ Division of Transplant Surgery, Virginia Commonwealth University, Richmond, Virginia, USA, vcu.edu

**Keywords:** analgesia, anesthesiology, hepatectomy, methadone, multimodal, pain, postoperative

## Abstract

**Background:**

Postoperative pain control is often suboptimal, with many patients experiencing inadequate relief or side effects. Methadone, a long‐acting μ‐opioid agonist and NMDA antagonist, provides stable analgesia and reduces postoperative opioid requirements when administered as a single intraoperative dose. However, its use has not been studied in living donor hepatectomy patients.

**Methods:**

We conducted a retrospective review of a quality improvement initiative involving 50 living donor hepatectomy patients treated from 2023 to 2025. 32 received usual opioid care and 18 received methadone, stratified into high‐dose (≥ 0.2 mg/kg lean body weight) and low‐dose (< 0.2 mg/kg) groups. The primary outcome was postoperative morphine milligram equivalents (MMEs) consumed during the hospital stay. Secondary outcomes included intraoperative MME, total MME, ICU and hospital length of stay, bowel recovery, and safety events.

**Results:**

Mean age was 39.4 years, 70% female and 72% white. Most patients (66%) underwent a minimally invasive, robotic surgical approach. Methadone was administered to 36%. Intraoperative opioid exposure was significantly higher in methadone groups compared with usual care (*p* < 0.0001), but postoperative opioid requirements did not increase. There was a trend toward lower postoperative morphine equivalents in patients receiving higher methadone doses (156.7 ± 152.8 vs. 260.4 ± 112.3, *p* = 0.09). No adverse events occurred; length of stay and bowel recovery were similar across groups.

**Conclusions:**

Intraoperative methadone administration in living donor hepatectomy appears safe and demonstrated a trend toward reduced postoperative MME use at higher doses of ≥ 0.2 mg/kg lean body weight. These findings warrant further prospective evaluation.

## 1. Introduction

Adequate postoperative pain management is of significant importance to patient care. Classically, this is achieved through a number of techniques, including epidural and intrathecal morphine, regional anesthesia such as transversus abdominis plane (TAP) catheters, and multimodal intravenous and oral medication approaches [1–3]. Despite this, many patients report inadequate pain relief and fluctuations in medication plasma concentration that can cause potential side effects [4].

Methadone is a long‐acting opioid that has recently regained significant attention in the field of anesthesiology for its ability to provide effective analgesia while reducing the risks and adverse effects associated with traditional opioid regimens or regional techniques. Methadone provides analgesic effects through stimulation of central μ‐receptors, coupled with n‐methyl‐d‐aspartate (NMDA) receptor antagonism, and has the longest elimination half‐life compared to other clinically used opioids such as fentanyl, morphine, or hydromorphone [5]. This unique pharmacology allows it to provide stable blood concentrations after a single intraoperative dose as opposed to shorter‐acting opioids that can cause patients to oscillate between analgesia and adverse effects [6].

Methadone has been utilized in numerous inpatient and outpatient surgical procedures including spine, colorectal, gynecological surgery, and others [4, 6–9]. In patients undergoing laparoscopic hysterectomies, those who received a single intraoperative methadone dose compared to short‐acting opioids experienced lower postoperative pain scores to discharge and consumed on average 9.44 mg fewer oral morphine equivalents. However, these benefits were deemed modest due to a smaller clinical significance than anticipated [4]. Dose‐finding studies in ambulatory and next‐day discharge surgeries found that an intraoperative methadone dose of 0.15–0.25 mg/kg ideal body weight produces effective analgesia and results in significantly less opioid use after discharge, indicating the long‐lasting benefit of methadone over traditional opioids [10, 11]. Furthermore, methadone has been studied in numerous surgical settings, including spinal fusion surgeries, hysterectomies, and cardiac surgery, and has consistently been associated with decreased postoperative opioid requirements [6]. However, methadone has not previously been studied in the living donor hepatectomy patient population.

In 2024, our institution transitioned away from neuraxial pain control after adopting a minimally invasive living donor hepatectomy surgical approach. The change led to the implementation of a quality initiative to improve postoperative pain management. The aim of this quality initiative was to determine the impact of intraoperative single‐dose methadone administration, as part of a perioperative pain management strategy, on postoperative opioid requirements.

## 2. Methods

This retrospective review evaluated the effects of intraoperative methadone administration on postoperative opioid use and recovery outcomes in living donor hepatectomy patients. Institutional approval for data acquisition and publication was granted as a quality improvement initiative (VCU IRB policy/HHS Common Rule 45 CFR 46.104(d)). The initiative met institutional criteria for quality improvement activities that do not constitute human subjects research, and standard informed consent was waived. This study was conducted in accordance with the ethical standards of the Declaration of Helsinki. Prior to initiation of this QI initiative, neuraxial anesthesia was abandoned secondary to a safety event, so the institutional standard of care was an opioid‐based analgesic. Prior to the quality initiative, methadone was not utilized; instead, patients typically received a fentanyl or hydromorphone PCA for 24 h before transitioning to oral opioids (oxycodone) and adjuncts (acetaminophen and methocarbamol).

**FIGURE 1:**
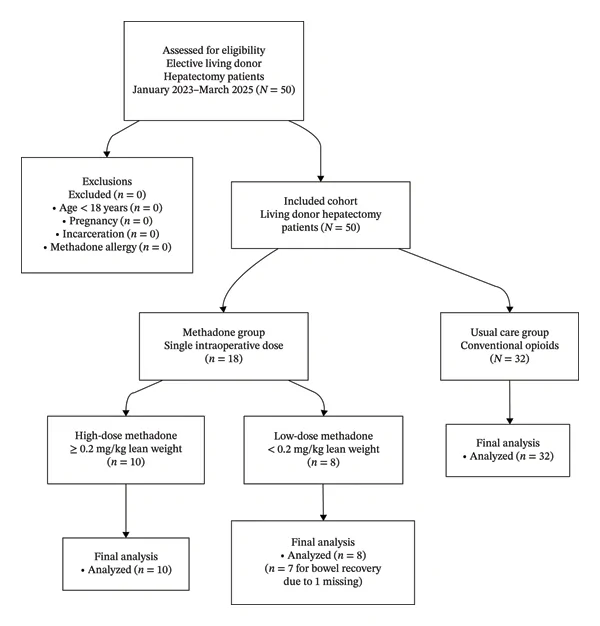
Flow diagram of patient selection, study eligibility, and group allocation.

Inclusion criteria included all patients who underwent elective living donor hepatectomy at Virginia Commonwealth University Medical Center in Richmond, Virginia, from January 2023 to March 2025. Exclusion criteria included patients less than 18 years old, pregnant patients, incarcerated individuals, and patients with a known allergy or contraindication to methadone. Given the nature of living liver donation, none of our patients met these exclusion criteria. Patient screening, eligibility assessment, exclusion criteria, and final group allocations are summarized in Figure 1. The study utilized electronic health records from VCU Hospital, encompassing 50 subjects: 32 subjects were in the usual care group, and 18 subjects were in the methadone administration group. We stratified the methadone administration group into a high methadone dose group (≥ 0.2 mg/kg lean body weight) and a low methadone dose group (< 0.2 mg/kg) to explore a potential effect of methadone dosage on postoperative and total morphine milligram equivalent (MME). Given the structure of this quality improvement initiative, all patients received inhalational general anesthesia without regional or neuraxial techniques, and no adjunct analgesics or sedatives (e.g., ketamine, lidocaine, antipsychotics, or dexmedetomidine) were utilized. The “usual care” group relied on conventional opioid regimens of fentanyl and hydromorphone, and doses were administered per practice norms and anesthesia team discretion. In the methadone group, methadone was administered as a single intravenous bolus during the peri‐induction period intraoperatively, prior to surgical incision, following conventions in existing literature. Dosing was calculated using lean body weight and was left to the discretion of the attending anesthesiologist within the institutional quality‐improvement framework (target range approximately 0.1–0.3 mg/kg lean body weight). Intraoperative or postoperative rescue fentanyl or hydromorphone was factored into total MME calculations.

The primary outcome of this study was postoperative MME consumed during the patient’s hospital stay. Secondary outcomes included intraoperative MME, total MME (combined intraoperative and postoperative), hospital length of stay, ICU length of stay, time to first bowel movement, and adverse safety events such as reintubations and need for non‐invasive mechanical ventilation.

We summarized the overall characteristics of subjects and by treatment group. Because treatment allocation and methadone dosing were determined by attending provider discretion rather than randomization, baseline clinical and demographic characteristics (including age, sex, race, BMI, and surgical approach) were evaluated across groups using ANOVA, Kruskal–Wallis, and chi‐square tests to assess for potential confounding and ensure group comparability (Table 1). We compared three groups with ANOVA for continuous variables: age in year and BMI. We used the Kruskal–Wallis test to test group difference for the length of stay (LOS), the length of ICU LOS days, time to bowel movement in days, methadone dose in mg/kg (total body weight), methadone dose in mg/kg (lean body weight), intraoperative MME, postoperative MME (calculated over the patient’s entire hospital stay), total (intraoperative and postoperative) MME in mg due to normality assumption violation for ANOVA. MME were calculated using a conversion factor of 13.5 (dosage × frequency × 13.5 = MME in mg/day) [12]. Pearson’s chi‐square test was used for categorical variables: race, methadone administration group (yes/no), and surgical approach (minimally invasive/open).

**TABLE 1:** Characteristics and perioperative outcomes stratified by intraoperative methadone dose.

Characteristic	Statistic	Usual care group *N* = 32	Doses ≥ 0.2 mg/kg *N* = 10	Doses < 0.2 mg/kg *N* = 8	*p*‐value
Age	(Mean, SD) < median, min, max>	(38.25, 9.15) < 38.50, 23.00, 61.00 >	(42.40, 12.33) < 41.50, 26.00, 59.00 >	(40.13, 11.10) < 40.50, 27.00, 59.00 >	0.6150
Gender					0.6495
Female	*n* (%)	21 (65.63%)	8 (80.00%)	6 (75.00%)	
Male	*n* (%)	11 (34.38%)	2 (20.00%)	2 (25.00%)	
Race					0.6634
Black or African American	*n* (%)	2 (6.25%)	0 (0.00%)	0 (0.00%)	
Unknown/other	*n* (%)	9 (28.12%)	2 (20.00%)	1 (12.50%)	
White	*n* (%)	21 (65.63%)	8 (80.00%)	7 (87.50%)	
BMI (kg)	(Mean, SD) < median, min, max>	(27.15,4.21) < 26.30, 19.40, 34.71 >	(26.87, 3.68) < 27.16, 20.32, 3453 >	(25.07, 3.62) < 24.88, 20.35, 31.00 >	0.4019
Surgical approach (open vs. minimally invasive)					0.3710
MINIMALLY INVASIVE	*n* (%)	20 (62.50%)	6 (60.00%)	7 (87.50%)	
OPEN	*n* (%)	12 (37.50%)	4 (40.00%)	1 (12.50%)	
Hospital days	(Mean, SD) < median, min, max>	(5.30, 2.02) < 5.00, 2.00, 12.00 >	(5.20, 1.44) < 5.50, 3.00, 7.00 >	(5.38, 1.71) < 5.50, 3.00, 8.00 >	0.9638
ICU days	(Mean, SD) < median, max>	(1.30, 0.67) < 1.00, 0.50, 4.00 >	(1.13, 0.32) < 1.00, 1.00, 2.00 >	(1.25, 0.71) < 1.00, 1.00, 3.00 >	0.7898
Time to bowel movement (days)	(Mean, SD) < median, min, max>	(3.44, 2.42) < 3.00, 0.00, 15.00 >	(3.30, 1.34) < 3.00, 1.00, 6.00 >	(4.63, 2.83) < 4.00, 2.00, 11.00 >	0.4334
Methadone dose (mg/kg) total body weight	(Mean, SD) < median, min, max>	(0.00, 0.00) < 0.00, 0.00, 0.00 >	(0.23, 0.04) < 0.23, 0.15, 0.30 >	(0.14, 0.03) < 0.13, 0.10, 0.19>	< 0.0001
Methadone dose (mg/kg) of lean body weight	(Mean, SD) < median, min, max>	(0.00,0.00) < 0.00, 0.00, 0.00 >	(0.31,0.08) < 0.32, 0.21, 0.46 >	(0.16, 0.02) < 0.16, 0.13, 0.18>	< 0.0001
Total intraop morphine equiv	(Mean, SD) < median, min, max>	(36.87, 16.52) < 39.00, 3.00, 72.00 >	(106.39, 24.47) < 108.00, 68.70, 141.00 >	(84.54, 21.89) < 76.50, 60.75, 117.00 >	< 0.0001
Total postop morphine equiv	(Mean,SD) < median, min, max>	(223.16, 175.15) < 211.65,0.75,950.70 >	(156.68, 152.83) < 118.75, 16.20, 549.25 >	(260.44, 112.25) < 243.30, 73.50, 436.50 >	0.0917
Total morphine equivalents	(Mean,SD) < median, min, max>	(260.03, 175.93) < 249.90, 39.75, 971.70 >	(263.07, 162.90) < 249.25, 84.90, 676.83 >	(344.99, 118.68) < 336.60, 154.50, 550.50 >	0.1186

All continuous variables were summarized as means with standard deviations and 95% confidence intervals, or medians with minimum and maximum. Categorical variables were reported as frequencies and percentages. Missing data were minimal; complete data were available for all 50 patients across primary and secondary MME outcomes, hospital length of stay, and ICU length of stay. Time to first bowel movement was missing for one patient (2%), who was excluded pairwise from that specific comparison. We checked linear regression model assumptions: linearity, normality, and homoscedasticity using multiple linear regression models and diagnostic plots (Supporting Tables 1–4 and Figures S1–S4). Given the retrospective quality‐improvement design and the fixed cohort size, a formal a priori sample size or power calculation was not performed. All statistical analyses were conducted using *R* 4.2.3, with a significance level at 0.05. The *R* package gtsummary Version 2.2.0 was used for the summary tables.

## 3. Results

Table 2 summarizes the characteristics of the study cohort (*N* = 50). The mean age was 39.38 years (SD = 10.06), and the majority of participants were female (70%) and White (72%). The mean BMI was 26.76 kg/m^2^ (SD = 4.02). Methadone was administered to 36% of patients. Most patients (66%) underwent a minimally invasive surgical approach. Median hospital stay was 5 days, and median ICU stay was less than 1 day. The median time to bowel movement was 3 days. The total intraoperative morphine equivalent dose (MED) had a mean of 58.40 (SD = 35.15), with a 95% CI of 48.41–68.39. Methadone dosing by lean body weight had a low mean of 0.09 mg/kg (SD = 0.13). No adverse safety events were reported such as reintubations or need for non‐invasive mechanical ventilation.

**TABLE 2:** Characteristics summary.

Characteristics (*N* = 50)	Mean, SD	Median, min, max	95% CI
Age	(39.38,10.06)	< 38.50, 23.00, 61.00 >	36.52, 42.24

*Gender*			
Female	35 (70.00%)		
Male	15 (30.00%)		

*Race*			
Black or African American	2 (4.00%)		
Unknown/other	12 (24.00%)		
White	36 (72.00%)		
BMI (kg)	(26.76,4.02)	< 26.47, 19.40, 34.71 >	25.62, 27.90

*Methadone (Y/N)*			
No	32 (64.00%)		
Yes	18 (36.00%)		

*Surgical approach (open vs. minimally invasive)*			
MINIMALLY INVASIVE	33 (66.00%)		
OPEN	17 (34.00%)		
Hospital days	(5.29, 1.84)	< 5.00, 2.00, 12.00>	4.75, 5.75
ICU days	(1.26, 0.61)	< 1.00, 0.50, 4.00 >	1.00, 1.43
Time to bowel movement (days)	(3.60, 2.32)	< 3.00, 0.00, 15.00 >	3.00, 3.50
Methadone dose (mg/kg) total body weight	(0.07, 0.10)	< 0.00, 0.00, 0.30 >	0.16, 0.22
Methadone dose (mg/kg) lean body weight	(0.09, 0.13)	< 0.00, 0.00, 0.46 >	0.18, 0.29
Intraoperative morphine equivalent	(58.40, 35.15)	< 53.69, 3.00, 141.00 >	45.30, 67.50
Postoperative morphine equivalent	(215.83, 163.01)	< 206.30, 0.75, 950.70 >	158.55, 237.50
Total morphine equivalent	(274.23, 165.65)	< 260.40, 39.75, 971.70 >	220.50, 298.17

*Note:* (Mean, SD) < median, min, max>; *n* (%). The three‐group comparison (usual care group = 32, high dose group = 10, low dose group = 8) shows no significant differences in age, gender, race, BMI, surgical approach, LOS, ICU stay, time to bowel movement, postoperative morphine equivalents, and total morphine equivalents, but there are highly significant differences in intraoperative morphine equivalents (*p* < 0.0001).

Abbreviation: CI = confidence interval.

## 4. Discussion

Effective pain control is a priority in living donor hepatectomy patients, as many patients experience inadequate relief and analgesic side effects that may negatively affect the donor experience [13, 14]. This quality initiative aimed to determine the impact of intraoperative single‐dose methadone administration in living liver donor patients, as part of a perioperative pain management strategy, on patient outcomes. In particular, this project intended to explore whether methadone had an impact on intraoperative, postoperative, and total morphine equivalents received.

The three groups, high‐dose methadone, low‐dose methadone, and control, were well balanced in regard to baseline characteristics with no significant differences in age, gender, race, BMI, or surgical approach. The study population was predominantly female across all groups, which may reflect the demographic distribution of patients undergoing this procedure at our institution.

There was found to be a statistically significant difference in total intraoperative morphine equivalents between the three groups. The high‐dose methadone group received the greatest amount of intraoperative morphine equivalents, with a mean of 106.39 ± 24.47 mEq, compared to 84.54 ± 21.89 mEq in the low‐dose methadone group and 36.87 ± 16.52 mEq in the usual care group. Higher intraoperative MMEs in the methadone groups were anticipated, as administration of a long‐acting opioid naturally increases the overall intraoperative opioid burden. Interestingly, the increased intraoperative opioids received with higher‐dose methadone did not correlate with increased postoperative opioids. This finding will be discussed further below.

There was a clinically significant difference in total postoperative MMEs between the dose‐stratified methadone groups, but this did not achieve statistical significance. The high‐dose methadone group required fewer morphine equivalents, 156.68 ± 152.83 MMEs, compared to the low‐dose group, 260.44 ± 112.25 MMEs (*p* = 0.09). Additionally, there was a trend toward lower MMEs when comparing the methadone group, without dose stratification, to control. The methadone group required 202.80 ± 142.72 MMEs as opposed to the control requiring 223.16 ± 175.15 MMEs (*p* = 0.66), although the difference was not statistically significant. This finding aligns with previous literature that has consistently found an intraoperative methadone dose is associated with decreased postoperative opioids across multiple surgical settings. In a randomized clinical trial of 120 adults undergoing tonsillectomy, a procedure frequently associated with severe postoperative pain, Bøndegaard et al. found that intraoperative methadone reduced cumulative postoperative opioid consumption and pain scores compared with intraoperative fentanyl [15]. In 156 randomized cardiac surgical patients given intraoperative methadone versus fentanyl, postoperative opioid requirements were reduced by 40% during the first 3 postoperative days in the methadone group. Similarly, in patients undergoing hysterectomies who received intraoperative methadone versus morphine, patients in the methadone group required fewer PACU opioids (2.0 mg vs. 4.4 mg). In spinal surgery patients, opioid requirements were 50% lower in the methadone group as compared to the group receiving continuous sufentanil infusion [6]. Furthermore, these individual findings are corroborated on a broader scale by a meta‐analysis that evaluates diverse surgical populations, including abdominal, cardiac, spine, and gynecological procedures [16]. This analysis demonstrated that intraoperative methadone significantly decreased postoperative opioid requirements across timeframes, with mean reductions of 8.42 mg MED at 24 h (*p* < 0.00001), 14.43 mg MED between 24 and 48 h (*p* < 0.00001), and 3.59 mg MED between 48 and 72 h (*p* = 0.007). The most pronounced opioid‐sparing benefit occurred during the critical 24–48 h window. Our findings add to the existing literature by demonstrating that intraoperative methadone is similarly associated with reduced postoperative opioid requirements in the unique setting of living donor hepatectomy. The observed signal toward lower postoperative morphine equivalents at higher methadone dose stratifications seems to support what is known about methadone’s dose‐dependent analgesic duration of action.

Although higher intraoperative methadone dosing was associated with higher intraoperative opioid administration, this did not translate into higher postoperative opioid requirements. Instead, we observed a trend toward reduced postoperative MMEs at higher intraoperative methadone doses, suggesting a possible “opioid sparing” effect. This finding is particularly relevant in light of concerns for tolerance development leading to decreased postoperative efficacy of opioids following intraoperative administration. The observed data suggest the opposite effect; while speculative, this could be due to methadone’s NDMA effects, which may mitigate central sensitization and “wind‐up” [17]. Although our study may have been underpowered to demonstrate statistical significance in postoperative MMEs in methadone versus control, the observed signal supports the safety and efficacy of intraoperative methadone administration in this setting. Additionally, while pain scores were not collected, postoperative MMEs were used as an objective surrogate measure for analgesic requirements following surgery.

There were no adverse safety events reported throughout the study, and the data also reveals no difference in length of stay or time to bowel movement among the three groups. While the median time to bowel movement was approximately 3 days, a slightly higher duration was observed in the low‐dose methadone group and is likely attributed to their higher total and postoperative MME requirements.

In our cohort, patients receiving higher intraoperative methadone doses (≥ 0.2 mg/kg lean body weight) demonstrated a nonsignificant trend toward lower postoperative MME requirements compared with lower‐dose methadone and usual care. While our observations align with prior dose‐finding studies that establish 0.15 mg/kg as the minimum effective floor for ambulatory surgery [10] and suggest analgesic benefits in the 0.2–0.3 mg/kg range for larger procedures [6, 11], our study was underpowered, and the difference did not achieve statistical significance. Therefore, these findings should be interpreted as hypothesis‐generating rather than definitive. Nevertheless, an intraoperative methadone dose in the 0.2–0.3 mg/kg lean body weight range appeared safe in this population and warrants prospective evaluation.

However, our study has several limitations inherent to its pragmatic, retrospective quality‐improvement design, which limits the ability to establish causality. First, because treatment allocation and specific methadone dosing were determined by attending provider discretion rather than randomization, selection bias and unmeasured baseline confounding remain possible. While key baseline characteristics were evaluated and confirmed to be well balanced across groups (Table 1), residual confounding cannot be completely excluded. Second, a formal a priori sample size or power calculation was not performed, and our modest sample size (*N* = 50) may have left our analysis underpowered to detect statistically significant differences in postoperative MME use or secondary outcomes. Furthermore, postoperative MME use was used as an objective surrogate for analgesic requirements. Standardized, prospectively collected pain scores were not available in the electronic health record for this quality‐improvement cohort; this absence is a recognized limitation and may limit direct comparison with trials that report numerical rating scale or visual analog scale data. Nevertheless, total postoperative MME is a reliable measure of analgesic needs, and large meta‐analyses show that intraoperative methadone lowers pain scores alongside reducing postoperative opioid requirements [16]. Additionally, perioperative regimens were not standardized, but variations were captured in the total MME calculations. Regional and neuraxial anesthesia techniques were underutilized at our institution, which may limit generalizability to other institutions with broader use of these techniques. Finally, while standard intraoperative electrocardiographic QT interval monitoring occurred in the operating room, monitoring was not performed outside of the operating room for the purposes of this quality initiative project. Thus, that data were not collected. Ultimately, our findings should be considered hypothesis‐generating and provide a strong foundation for future randomized prospective trials to clarify the relationship between methadone dosage and postoperative opioid requirements in the living donor hepatectomy patient population.

## 5. Conclusion

Intraoperative methadone administration in living donor hepatectomy appears safe and was associated with a nonsignificant trend toward reduced postoperative MME requirements, particularly at higher doses of ≥ 0.2 mg/kg lean body weight. We also observed a trend toward earlier time to bowel movement in the higher‐dose methadone group. While our findings are consistent with prior literature suggesting the analgesic benefits with higher‐dose methadone, these results are hypothesis‐generating and provide a strong foundation for future prospective, randomized controlled trials with standardized dosing to definitively characterize the relationship between methadone usage and postoperative opioid requirements.

## Author Contributions

All authors met the ICMJE criteria for authorship. Specific contributions are as follows:

Adam Seibert, MD: contribution to study conception/design, data acquisition, data analysis/interpretation, drafting of the manuscript, and critical revision of the manuscript. Devika Puri, BS: contribution to data acquisition, data management, drafting of the manuscript, and critical revision of the manuscript. Zachary Whong, BS: contribution to data acquisition, data management, drafting of the manuscript, and critical revision of the manuscript. Beomchang Kim, MS: contribution to formal statistical analysis and data interpretation, and critical revision of the manuscript. Rasha Alsaadawi, PhD: contribution to formal statistical analysis and data interpretation, and critical revision of the manuscript. Le Kang, PhD: contribution to formal statistical analysis and data interpretation, and critical revision of the manuscript. Christin Kim, MD: contribution to study design, protocol oversight, and critical revision of the manuscript. Seung Duk Lee, MD: contribution to study design, patient selection, and critical revision of the manuscript. Vinay Kumaran, MD: contribution to study design, patient screening, and critical revision of the manuscript. Smita Pancholia, MD: contribution to study conception/design, data acquisition, drafting of the manuscript, and critical revision of the manuscript. Sergio Navarrete, DO: contribution to study conception/design, data acquisition, data analysis/interpretation, drafting of the manuscript, and critical revision of the manuscript.

## Funding

This project was supported in part by the Biostatistics, Epidemiology and Research Design (BERD) core of the C. Kenneth and Dianne Wright Center for Clinical and Translational Research (CTSA award UM1TR004360 from the National Center for Advancing Translational Sciences).

## Disclosure

The contents are solely the responsibility of the authors and do not necessarily represent official views of the National Center for Advancing Translational Sciences or the National Institutes of Health.

## Conflicts of Interest

The authors declare no conflicts of interest.

## Supporting Information

Additional supporting information can be found online in the Supporting Information section.

## Supporting information


**Supporting Information** Supporting information associated with this article, including diagnostic plots and regression model summaries (Tables 1–4 and Figures S1–S4), is available online. Initial multivariable linear regression models evaluated the impact of intraoperative methadone administration and hospital length of stay (LOS) on intraoperative and postoperative morphine milligram equivalents (MME). Univariable models (Tables 2 and 4) were subsequently fit after removing nonsignificant predictors (LOS for intraoperative MME; methadone group for postoperative MME). Diagnostic plots (Figures S1–S4) confirmed that the statistical assumptions of linearity, normality, and homoscedasticity were adequately satisfied for all primary and secondary regression analyses.

## Data Availability

The data that support the findings of this study are available from the corresponding author upon reasonable request.
