# Supplementary material for: Intraoperative Methadone for Postoperative Pain Control in Living Donor Hepatectomy
Source: J Transplant. 2026 Sep 9;2026:8206764. doi: 10.1155/joot/8206764 (PMC13555282; doi:10.1155/joot/8206764)
Supplement: Supplementary file 1 [file JOOT-2026-8206764-s001.docx]

**Supplementary material (Tables 1-4, Figures S1-S4)**

We first fit a multiple linear regression model to evaluate the impact of methadone administration on Intraoperative MME, while accounting for LOS. The result indicated that LOS had no statistically significant effect on the model (Table 1). Therefore, we refit the model without including LOS. Table 2 presents the model summary. Similarly, a multiple linear regression model was used to evaluate the impact of methadone administration on Postoperative MME, while accounting for LOS. The result indicated that the methadone administration group had no statistically significant effect on the model (Table 3). Therefore, we refit the model excluding the methadone administration group. Table 4 presents the model summary. The plots (Fig S1-S4) indicated that the assumptions of normality and homoscedasticity were adequately satisfied for our analyses.

**Table 1.** Linear Regression Results for Intraoperative MME by Methadone Administration Group and Length of Stay (model 1)

**Table 2.** Linear Regression Results for Intraoperative MME by Methadone Administration Group (model 2)

**Table 3.** Linear Regression Results for Postoperative MME by Methadone Administration Group and Length of Stay (model 3)

**Table 4.** Linear Regression Results for Postoperative MME by Length of Stay (model 4)

**Figure S1.** Model diagnostics for model 1

(Top left: linearity, Top right: normality, Bottom left: homoscedasticity)

**Figure S2.** Model diagnostics for model 2

(Top left: linearity, Top right: normality, Bottom left: homoscedasticity)

**Figure S3.** Model diagnostics for model 3

(Top left: linearity, Top right: normality, Bottom left: homoscedasticity)

**Figure S4.** Model diagnostics for model 4

(Top left: linearity, Top right: normality, Bottom left: homoscedasticity)
